# Genetic Landscape of Male Breast Cancer

**DOI:** 10.3390/cancers13143535

**Published:** 2021-07-15

**Authors:** Fernando Augusto Batista Campos, Etienne Rouleau, Giovana Tardin Torrezan, Dirce Maria Carraro, José Claudio Casali da Rocha, Higor Kassouf Mantovani, Leonardo Roberto da Silva, Cynthia Aparecida Bueno de Toledo Osório, Solange Moraes Sanches, Sandrine M. Caputo, Elizabeth Santana dos Santos

**Affiliations:** 1 Deparment of Medical Oncology, A.C.Camargo Cancer Center, Sao Paulo 01509-010, Brazil; solange.sanches@accamargo.org.br (S.M.S.); elizabeth.santos@accamargo.org.br (E.S.d.S.); 2 Department of Medical Biology and Pathology, Gustave Roussy, Cancer Genetics Laboratory, Gustave Roussy, 94805 Villejuif, France; etienne.rouleau@gustaveroussy.fr; 3 Genomics and Molecular Biology Group, International Center of Research CIPE, A.C.Camargo Cancer Center, Sao Paulo 01509-010, Brazil; giovana.torrezan@accamargo.org.br (G.T.T.); dirce.carraro@accamargo.org.br (D.M.C.); 4 National Institute of Science and Technology in Oncogenomics (INCITO), Sao Paulo 01508-010, Brazil; 5 Department of Oncogenetics, A.C.Camargo Cancer Center, Sao Paulo 01509-010, Brazil; casali.rocha@accamargo.org.br; 6 Department of Obstetrics and Gynecology, Faculty of Medical Sciences, State University of Campinas (UNICAMP), Campinas 13083-881, Brazil; higor@unicamp.br (H.K.M.); lrs@unicamp.br (L.R.d.S.); 7 Department of Pathology, A.C.Camargo Cancer Center, Sao Paulo 01509-010, Brazil; cabtoledo@accamargo.org.br; 8 Department of Genetics, Institut Curie, 75248 Paris, France; sandrine.caputo@curie.fr; 9 Institut Curie, PSL Research University, 75005 Paris, France; 10 Centro de Oncologia, Hospital Sírio Libanês, Sao Paulo 01308-050, Brazil

**Keywords:** male breast cancer, *BRCA2*, *BRCA1*, hereditary breast cancer, genetic testing

## Abstract

**Simple Summary:**

Male breast cancer is a rare disease, representing around 0.5% of the malignances in men. Although they receive the same treatment as women with breast cancer, there is increasing knowledge showing that both have a distinct genetic background. Pathogenic variants in cancer predisposing genes are a likely etiology for male breast cancer in 4% to 40% of the cases, and it is currently recommended that all men diagnosed with breast cancer be offered genetic counseling followed by genetic testing. Even though, men are still less likely to undergo the test than women for many reasons, which include an unfamiliarity with the issue by health professionals. The purpose of this article is to review the current knowledge of the germline genetic background of male breast cancer and its impact in the management of the patients and their families.

**Abstract:**

Male breast cancer (MBC) is now considered molecularly different from female breast cancer (FBC). Evidence from studies indicates that common genetic and epigenetic features of FBC are not shared with those diagnosed in men. Genetic predisposition is likely to play a significant role in the tumorigenesis of this rare disease. Inherited germline variants in *BRCA1* and *BRCA2* account for around 2% and 10% of MBC cases, respectively, and the lifetime risk of breast cancer for men harboring *BRCA1* and *BRCA2* mutations is 1.2% and 6.8%. As for FBC, pathogenic mutations in other breast cancer genes have also been recently associated with an increased risk of MBC, such as *PALB2* and *CHEK2* mutations. However, while multigene germline panels have been extensively performed for BC female patients, the rarity of MBC has resulted in limited data to allow the understanding of the magnitude of risk and the contribution of recently identified moderate penetrance genes of FBC for MBC predisposition. This review gathers available data about the germline genetic landscape of men affected by breast cancer, estimated risk associated with these genetic variants, and current guidelines for clinical management.

## 1. Introduction

Male breast cancer (MBC) is a rare disease, corresponding to less than 1% of all cases of breast cancer (BC) and about 0.5% of the malignancies in men in Western countries [1,2,3]. The approximate lifetime risk of BC for a man is 1:1000, whereas it is 1:8 for a woman [4]. Over the past decades, an increase on the incidence of the disease has been observed, partly related to the higher rates of obesity, a well-known risk factor for BC, and also to the increasing longevity of the population [5,6,7,8,9]. This was accompanied by a greater interest from the scientific community in broadening the understanding of the tumorigenesis of BC in men, and it seems to be clear that the vast knowledge generated so far about female breast cancer (FBC) cannot be completely transposed to MBC [10]. Despite this, especially due to the rarity of the disease and the difficulty in designing clinical trials focused on this population, as well as the underrepresentation of men on existing studies [11], the treatment of MBC still follows the same recommendations for FBC [12]. 

Genetics plays an important role in predisposition to MBC. Pathogenic variants (PVs) in *BRCA2* are one of the most remarkable risk factors. Furthermore, studies with multigene panel tests (MGPT) have identified alterations in other genes suspected to increase the risk for MBC, and depending on the studied population, the detection rate of PVs in cancer related genes is as high as 32% [13]. Currently, it is strongly recommended that every man with BC be offered genetic counseling followed by genetic testing for high-penetrance germline PVs in BC susceptibility genes, irrespective of family history of cancer [14]. Notwithstanding, men are still less likely to undergo the test for many reasons, which range from individual and psychological factors to unfamiliarity with the issue by the health professionals [15,16,17]. 

This review focuses on the contribution of hereditary predisposition for MBC and its genetic background. Deeper insight on the carcinogenesis of this rare disease may provide patients and their relatives greater access to personalized therapies and individualized surveillance. 

## 2. Epidemiology and Non-Genetic Risk Factors for MBC

The number of newly diagnosed MBC cases increased worldwide from 8500 in 1990 to 23,100 in 2017, according to data from the Global Burden of Disease 2017 database [18]. In 2021, about 2650 men are expected to be diagnosed with BC in the US, with an estimated 530 deaths due to the disease [1]. Its incidence, however, varies across the globe, with lower rates in Asia and higher in Africa, where the male-to-female BC ratio was found to be 0.042, while it is around 0.01 in other populations [19]. This fact may be attributed to the high prevalence of endemic infectious diseases, such as schistosomiasis and hepatitis B/C, which course with liver damage and consequent hyperestrogenism, a known risk factor for MBC [20]. Regardless of geographical location, Black men are more likely to develop BC than White men (incidence of 1.8 per 100,000 vs. 1.1 per 100,000, respectively) [21]. It has also been previously reported a higher incidence of BC in Jewish men (2.3/100,000 per year), for whom the spectrum of germline PVs in *BRCA1* and *BRCA2* (*BRCA1/2*) encloses few predominant and recurrent PVs, both in Jewish Ashkenazi and in non-Ashkenazi [22,23,24,25]. 

In comparison with women, men with BC tend to be diagnosed at an older age, for both early and advanced disease, with a median age at diagnosis ranging between 60 and 70 years, compared to 55–60 years in women [12,26,27,28]. Black men are more likely to be younger when diagnosed and have worse survival rates than their White counterparts [29,30,31]. A clear progress in BC survival has been seen in the last decades, but the rate of improvement is slower for men compared with that for women [28]. 

Male and female BC patients share some similar risk factors, such as advanced age and family history [5,28]. Furthermore, as in FBC, emerging data shows higher frequency of MBC with increasing levels of estradiol and elevated estrogen/androgen ratio [32]. An example of that is Klinefelter syndrome (KS), the most common chromosomal abnormality in men, resulting in a 47,XXY karyotype [33]. This syndrome is characterized by hypogonadism, and patients can present testicular dysgenesis, gynecomastia, learning difficulties, and infertility [33]. The increased estrogen/testosterone ratio observed in these individuals may explain their 20- to 50-fold greater risk of BC [33,34,35,36]. 

Conditions associated to elevated estrogen levels like obesity, gynecomastia, and liver disease, as well as the use of exogenous estrogens (i.e., estrogen treatment for prostate cancer and estrogen therapy for male-to-female transsexuals) or exogenous testosterone (i.e., hormonal treatment for male hypogonadism and testosterone therapy for transgender males) have also been linked with increased risk of MBC [37,38,39,40,41,42,43]. In addition, lack of physical activity, as well as exposure to radiation and organic solvents, are also supposed to represent risk factors for MBC [40,44,45].

## 3. MBC Disease Characteristics

At diagnosis, men with BC tend to present a more advanced stage of the disease when compared with women, usually with tumors >2.0 cm and positive axillary lymph nodes [26,27]. Many factors can explain the late diagnosis in men: absence of screening programs, lack of awareness about the condition by male population, embarrassment due to a stigmatization of the disease, and misjudgment by primary care physicians [20,46,47,48]. Bilateral disease at diagnosis is very rare [49]. More than 80% of the cases are invasive ductal carcinomas, followed by in situ ductal carcinomas [50,51]. Lobular invasive carcinoma is rarely encountered in men compared with women (1.2% vs. 8.2%), whereas papillary histology is more common in men (4.4% vs. 0.7%) [52]. The majority of MBC present histologic grade 2 and are hormone receptor-positive (HR+), including the androgen-receptor [50,51]. Human epidermal growth factor receptor-2 (HER2)-positive and triple-negative BC are rare, representing about 10–15% and 3–5% of the cases, respectively [53,54,55].

In 2018, Liu et al. [56] published a study including data from 289,673 patients registered in the Surveillance, Epidemiology, and End Results (SEER) program from 2005 to 2010 with a diagnosis of BC, of whom 0.71% were men. The 5-year overall survival (OS) was 82.8% for men, which was lower than the 88.5% for women. Additionally, the risk of death for men with BC was 43% higher than for women (hazard ratio (HR) 1.43; 95% confidence interval (CI), 1.26–1.61). 

Advanced stage at diagnosis does not solely explain the worse prognosis of MBC. A study including only patients with stages I and II BC showed 5- and 10-year OS rates of 82% and 61% for men, while it was 90% and 79% for FBC, respectively [57]. There might be other gender-related factors and unique biological features, as well as unknown underlying mechanisms of the disease not explored in the treatment responsible for the higher rate of death in men [56]. The study by Massarweh et al. [52] evaluated the 21-gene breast Recurrence Score (RS) results of men and women with HR+/HER2-negative BC and showed that men presented significantly high ≥31 RS than women (12.4% vs. 7.4%). Considering only the high-risk group, MBC patients had worse 5-year OS and 5-year breast cancer-specific survival compared with women (69.9% vs. 89.9%, and 81.1% vs. 94.9%, respectively).

Some clinical and pathological characteristics are associated with worse prognosis in MBC. Recently, Yadav et al. [54] published one of the largest studies regarding MBC, including 10,873 men diagnosed with BC stages I–III. A multivariate analysis showed the following was associated with worse OS: older age, Black race, higher Charlson Comorbidity Index, high tumor grade, advanced stage, and undergoing total mastectomy. Other studies showed that HER2-positive and triple-negative tumors are also associated with shorter survival [53]. 

## 4. Genomic and Epigenomic Landscape of MBC

**Figure 1:**
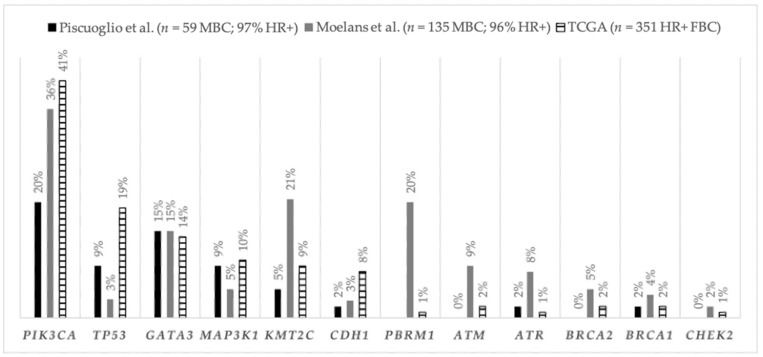
Frequency of the altered genes in luminal tumors from MBC and from breast cancer TCGA samples. First are percentages of the most common altered genes in the studies. Last are presented the frequencies of the most common altered DNA repair-related genes, besides *TP53*, which include *ATM*, *ATR*, *BRCA2*, *BRCA1*, and *CHEK2*.

The genomic and epigenomic landscape of MBC is not as extensively characterized as FBC. In the first breast study from the Cancer Genome Atlas (TCGA) program, only six out of 825 samples were from MBC patients [58]. Significant differences in the frequency of tumor mutations were observed according to the subtype of BC in the cohort, notably genetic heterogeneity within luminal BCs. Such diversity was also reported in two studies focusing on the male population with BC (Figure 1). 

The study by Piscuoglio et al. [59], encompassing 59 MBC cases, of which 97% were ER-positive/HER2-negative tumors (71% luminal B-like), sequenced 241 genes most prevalent in FBC, demonstrating that *PIK3CA*, *GATA3*, *TP53*, and *MAP3K1* were significantly mutated. In the same fashion as for FBC, differences in the frequencies of mutated genes within each luminal subgroup were found, with *PIK3CA*, *HER2C2*, and *MAP3K1* being the most frequently mutated genes in luminal A-like MBC (all at 12%), whereas *PIK3CA* (24%) and *GATA3* (21%) were the most frequently mutated in luminal B-like MBC. Of note, mutations in DNA repair genes were more frequent within luminal B-like MBC than luminal A-like MBC (33% and 6%, respectively; *p* = 0.04). Another study by Moelans et al. [60], including 135 MBC cases (96% luminal subtype), confirmed the high prevalence of *PIK3CA* and *GATA3* mutations and, also, demonstrated the high frequency of *PBRM1* and *KMT2C* PVs in this population. In the TCGA BC program, two MBC harbored *PIK3CA* PVs, and no *TP53* PVs were identified [58].

Taken together, findings from both studies show that, similarly to luminal FBC, luminal MBC presents significantly recurrent mutated genes such as *TP53*, *MAP3K1*, and *PIK3CA* and a higher frequency of mutations in genes associated with DNA repair pathways. Interestingly, some studies have described the *PIK3CA* PVs hotspots identified in MBC to be different from those found in FBC, which was also seen for *GATA3*-mutated hotspots, suggesting a gender effect [59,61,62]. Others found no such differences in their cohorts [60,62]. 

Johansson et al. [63] described the existence of two subgroups of MBC based on transcriptional and copy number profiling findings and designated luminal M1 and luminal M2, which did not correlate with the intrinsic subtypes described for FBC. The luminal M1 subgroup was characterized by a complex range of chromosomal aberrations and upregulation of the genes involved in cell migration, cell division, and angiogenesis, while the luminal M2 subgroup showed the upregulation of immune-related and ER pathway-associated genes. In another study, the same author used an integrated array comparative genomic hybridization and gene expression data to analyze driver genes in FBC and MBC samples [64]. The work demonstrated remarkable gender differences. Somatic mutations in three known cancer genes were identified in MBC, *MAP2K4*, *LHP*, and *ZNF217*, as well as in two new genes involved in proliferation, invasion, and metastasis (*THY1* and *SPAG5*). Only two candidate driver genes were shared between FBC and MBC (*TAF4* and *CD164*), and even when they were analyzed separately according to the intrinsic subgroups of FBC, just three more candidate drivers common within them were found (*ARHGAP30*, *COG3*, and *SPAG5*), suggesting that MBC may display different intrinsic subgroups. Other studies have also been able to identify distinct gender-related landscapes of somatic alterations [65,66].

Similarly to FBC, MBC is characterized by a range of copy number variations (CNVs) [67,68]. In the study by Moelans et al. [60] previously described, each tumor harbored, on average, 138 gene CNVs, including amplifications and homozygous deletions. Only 40% of amplified genes in MBC were the same as in FBC. Other studies corroborated these gender differences, including more whole chromosome arm gains, fewer losses, and high-level amplifications in MBC when compared to FBC [69]. Two chromosomal regions, including the known oncogenes (*ZNF282, PAK1, RSF1*, and *GAB2*), were amplified exclusively in MBC. Additionally, frequent gains were observed in *EGFR* and *CCDN1*, and common losses in *BRCA2, PALB2, EMSY, CHEK1, CPD*, and *CHEK2* [70,71]. Copy number gains were associated with a more aggressive phenotype, and the *CCDN1* amplification was the most important prognostic factor for worse outcomes in MBC [70]. 

Besides the genomic alterations, different epigenetic phenomena also play a role in MBC tumorigenesis. They are frequently an early event in cancer development and are associated with poorer prognosis. A study by Kornegoor et al. [72] applied a methylation-specific multiplex ligation-dependent probe amplification (MLPA) technique and found that more than half of the 108 MBCs samples showed promoter hypermethylation in the genes related to DNA repair mechanisms, cell adhesion, growth, and migration, among other functions (*MSH6, WT1, PAX5, CDH13, GATA5*, and *PAX6*). This finding was similar to what is seen in FBC. However, hypermethylation in *ESR1, BRCA1, BRCA2, PTEN, STK11, VHL*, and *ATM* was more common in FBC. When analyzing the cumulative methylation index (CMI), calculated as the sum of the methylation percentage of all the genes, the study showed that MBC tumors with high CMI (>350) had worse survival (*p* = 0.048; HR 2.5). These findings highlighted the differences in carcinogenesis between FBC and MBC, but further studies are necessary to validate the methylation status in such genes in order to be useful as a potential target in clinical practice.

Interestingly, it seems that epigenetic regulation of the ER pathway might differ between men and women. Pinto et al. [73] studied epigenetic alterations in 56 familial BC patients (27 males and 29 females), showing that a higher methylation and lower expression of *RASSF1A* (a gene linked to the downregulation of ER-alpha) resulted, contradictorily, in an absence of ER expression in MBC, which did not happen in FBC. 

In addition to methylation, as for FBC, microRNA (miRNA) epigenetic alterations are also common in MBC but with a distinct miRNA signature [73,74,75]. 

The epigenetic signatures of *ATM*, *BRCA1*, *PALB2*, *RAD51B*, and *XRCC3* have also been explored in MBC (32.4% with germline *BRCA2* PV) in matched normal and gynecomastia tissue samples (obtained from patients without cancer or a family history of BC) [76]. Remarkably, the methylation panel in tissue samples combining *RAD51B* and *XRCC3* accurately discriminated MBC from gynecomastia. Interestingly, normal breast tissues disclosed *RAD51B* and *XRCC3* promoter methylation at lower levels compared to BC, suggesting the existence of a cancerization field effect. 

## 5. Inherited Pathogenic Variants Contribution to MBC Development

Studies in MBC have revealed an evident family clustering of some cases and the contribution of the genes initially identified in the female population as risk factors for MBC as well. Indeed, PVs in *BRCA1*, and mainly in *BRCA2*, were the first to be associated with an increased risk of BC in men [77,78]. MGPT has contributed to the identification of cancer predisposing genes associated with the development of the disease in men, showing that the germline alterations contributing to MBC are similar to those related to FBC, including PVs in high- or moderate-penetrance BC genes, as well as more common low-penetrance variants [25]. Depending on the studied population, PVs in cancer-related genes can be detected in up to a third of cases. Currently, PVs in two high-penetrance BC genes, mainly *BRCA2* but also *BRCA1*, and other two moderate-penetrance BC genes, *PALB2* and *CHEK2*, are established as genetic risk factors for MBC, with emerging evidence for the *ATM* gene [44,79].

### 5.1. BRCA2 and BRCA1

*BRCA1* and *BRCA2* (*loci* 17q21 and 13q12.3, respectively) are major tumor-suppressor genes involved in DNA double-stranded break repair by homologous recombination (HRR) [78,80]. Since their identification, *BRCA1/2* genes were linked to BC predisposition. PVs in *BRCA1/2* show high penetrance and are distributed in an autosomal-dominant pattern among carriers, resulting in a significantly elevated risk also of other malignancies, such as ovarian, prostate, melanoma, and pancreatic cancer [81,82]. The prevalence of germline *BRCA1/2* PVs in men and women with BC unselected for family history, age, ethnicity, or molecular subtype is estimated to range from 2.7% to 6.1% [83]. 

Different from FBC, *BRCA2* gene is much more frequently altered in men than *BRCA1* [84]. In high-risk BC families, *BRCA2* PVs are responsible for 60–70% of MBC cases [79]. The estimated lifetime risk of BC is 5–10% among male *BRCA2* PV carriers, compared to a 0.1% risk in the general population [85,86,87]. Germline PVs frequencies in the *BRCA2* gene in MBC cohorts vary from 3.7% to 40%, depending on the characteristics of the studied population and their ancestral background [13,79,88,89,90,91,92,93,94,95,96,97]. As it has been shown in FBC, the MBC phenotype is peculiar in men harboring *BRCA1*/*2* PVs (Table 1). *BRCA2* MBC commonly show high tumor grades, do not express progesterone receptors, and are enriched in HER2. Additionally, an age-specific association is found, with men younger than age 50 years more frequently presenting grade 3 tumors [98]. 

On the other hand, the frequency of *BRCA1* PVs is low in unselected populations of MBC, ranging from 0% to 5% [13,79,88,89,90,91,92,93,94,95,96,97]. It is higher in populations in which a founder PV exists (10–16%) [79]. *BRCA1*-associated MBC is usually high-grade hormonal receptor-positive and HER2-negative [99,100,101]. 

Some differences can be seen in comparison to FBC, in which *BRCA1*-related tumors tend to be triple-negative, which is not observed in *BRCA1*-MBC. Additionally, both *BRCA1*- and *BRCA2*-related FBC are usually diagnosed at a younger age than in the general population, which is not the case for *BRCA1/2* men with BC [98,99,100,101,102]. Moreover, in contrast to FBC, family history may not be a strong predictor of a *BRCA1/2* PV in men with BC [103]. 

**Table 1:** Selected studies evaluating the association between clinic–pathological characteristics and the *BRCA1/2* status in MBC.

	*BRCA1* Pathogenic Variant			*BRCA2* Pathogenic Variant			*BRCA* Wild-Type		
Author, year (total participants)	Ottini 2012 (378)	Deb 2012 (60)	Gargiulo 2016 (47)	Ottini 2012 (378)	Deb 2012 (60)	Gargiulo 2016 (47)	Ottini 2012 (378)	Deb 2012 (60)	Gargiulo 2016 (47)
Number of patients/Total evaluated (%)	4/378 (1.1)	3/60 (5)	1/17 (5.9)	46/378 (12.2)	25/60 (41.6)	5 (29.4)	328/378 (86.7)	32/60 (53.3)	10/17 (58.8)
Mean/Median age (years)	62.0/NI	NI/65.6	NI/40.0	58.9/NI	NI/61.0	NI/72	NI/NI	NI/63.2	NI/61.0
FH of breast/ovarian cancer (%)	3 (75)	3 (100)	1 (100)	31 (67.4)	25 (100)	2 (40)	105 (32)	32 (100)	5 (50)
Personal history of other cancers (%)	0	0 (0)	1 (100)	12 (26.1) ^1^	5 (8.3) ^2^	2 (40)	44 (13.4)	5 (8.3)	2 (20)
Contralateral BC (%)	0	0 (0)	NI	7 (15.2)	1 (1.6)	NI	9 (2.7)	1 (1.6)	NI
Histology (%)	(*n* = 4)	(*n* = 3)	(*n* = 1)	(*n* = 34)	(*n* = 25)	(*n* = 5)	(*n* = 254)	(*n* = 34)	(*n* = 10)
Invasive ductal carcinoma	4 (100)	2 (66.6)	1 (100)	30 (88.3)	24 (96)	5 (100)	220 (86.6)	30 (88.2)	10 (100)
In situ ductal carcinoma	0 (0)	0 (0)	0 (0)	2 (5.9)	0 (0)	0 (0)	20 (7.9)	0 (0)	0 (0)
Invasive medullary carcinoma	0 (0)	0 (0)	0 (0)	1 (2.9)	0 (0)	0 (0)	0 (0)	0 (0)	0 (0)
Invasive lobular carcinoma	0 (0)	0 (0)	0 (0)	0 (0)	0 (0)	0 (0)	4 (1.6)	2 (6.3)	0 (0)
Others	0 (0)	1 (33.3)	0 (0)	1 (2.9)	1 (4)	0 (0)	10 (3.9)	2 (6.3)	0 (0)
Molecular characteristics (%)	(*n* = 4)	(*n* = 3)	(*n* = 1)	(*n* = 19)	(*n* = 25)	(*n* = 5)	(*n* = 166)	(*n* = 30)	(*n* = 9)
HR-positive	3 (75)	3 (100)	1 (100)	16 (84.2)	23 (92.0)	5 (100)	159 (95.8)	26 (86.7)	9 (100)
HER2-positive	0 (0)	0 (0)	0 (0)	3 (15.8)	2 (8.0)	1 (20)	1 (0.6)	3 (10.0)	1 (11)
Triple-negative	1 (25)	0 (0)	0 (0)	0 (0)	0 (0)	0 (0)	6 (3.6)	1 (3.3)	0 (0)
Histological grade (%)	(*n* = 3)	(*n* = 3)	NI	(*n* = 31)	(*n* = 25)	NI	(*n* = 227)	(*n* = 32)	NI
1 to 2	1 (33.3)	0 (0)		14 (45.2)	13 (52)		169 (74.4)	20 (62.5)	
3	2 (66.7)	3 (100)		17 (54.8)	12 (48)		58 (25.6)	12 (37.5)	
Staging (%)	(*n* = 3)	NI	(*n* = 1)	(*n* = 24)	NI	(*n* = 4)	(*n* = 187)	NI	(*n* = 10)
I–II	2 (66.7)		1 (100)	15 (62.5)		2 (40)	151 (80.7)		9 (90)
III–IV	1 (33.3)		0 (0)	9 (37.5)		2 (40)	36 (19.3)		1 (10)
Node status, positive (%)	(*n* = 3)	(*n* = 3)	(*n* = 1)	(*n* = 30)	(*n* = 20)	(*n* = 5)	(*n* = 209)	(*n* = 23)	(*n* = 10)
	2 (66.7)	2 (66.7)	1 (100)	17 (56.7)	9 (45)	2 (40)	80 (38.3)	9 (39.1)	5 (50)

NI: not informed, FH: family history, BC: breast cancer, HR: hormonal receptor, and HER2: human epidermal growth factor receptor 2. ^1,2^: mainly prostate cancer (58% and 50%, respectively).

### 5.2. PALB2

*PALB2*, the partner and localizer of *BRCA2* and the bridge between *BRCA1/2*, located in 16p12.2, is a tumor-suppressor gene that encodes a protein essential for homologous recombination in collaboration with *BRCA2* during DNA double-stranded break repair [104]. Historically, Silvestri et al. [105] reported the first insights about *PALB2* predisposition to MBC. Among 97 *BRCA1/2*-negative men with BC submitted to the genetic sequencing of this gene, no PV was initially found. Later, the same authors published their findings of germline whole-exome sequencing and targeted gene sequencing in 48 high-risk MBC patients, all *BRCA1/2*-negative, and identified a *PALB2* PV in one of them. This can suggest that men with BC who are negative for *BRCA1/2* PVs and have a strong family history of BC should proceed in investigating the status of *PALB2* [106].

In 2017, Pritzlaff et al. [92] reported a cohort of 715 MBC patients referred for multigene panel testing and found PVs in *PALB2* in 0.8% of 621 tested and a significant high risk for MBC (OR = 6.6). Recently, Yang et al. [107] analyzed data from 524 families with *PALB2* PVs, describing a risk association of 7.34 for MBC (95% CI, 1.28–42.18) with an estimated lifetime risk of 1% (95% CI, 0.2–5%). 

There is evidence supporting that *PALB2*-associated FBC, specifically those harboring *PALB2* c.1592del (p.Leu531fs) PV, displays aggressive clinicopathological features, such as the absence of hormonal receptors expression, advanced stage at diagnosis, and high Ki67 expression. However, there is no evidence yet of perhaps a more aggressive behavior concerning MBC [104]. 

### 5.3. CHEK2

Mutations in cell cycle checkpoint kinase 2 (*CHEK2*) were first associated with an increased risk of BC in women and men in 2002 [108]. *CHEK2* (locus 22q12.1) encodes a cell cycle checkpoint kinase activated through phosphorylation by ataxia telangiectasia mutated (*ATM*) in response to double-stranded DNA breaks, playing an important role in the DNA HRR pathway [109]. In that first report, the recurrent PV *CHEK2* c.1100del (p.Thr367Metfs*15) was found to confer an approximately two-fold increase of BC risk in women and a 10-fold increase of risk in men [108]. Although some subsequent studies could not establish *CHEK2* c.1100del as a risk allele for MBC due to the low number of patients and lack of statistical power [110,111,112,113], a recent meta-analysis confirmed its association with the increased risk of MBC, although substantially inferior to what was initially suggested (OR = 3.13, 95% CI 1.94–5.07) [114]. 

Few studies have described the clinicopathological characteristics of *CHEK2*-related MBC. Hallamies et al. [115] reported that four out of 68 men with BC were found to harbor *CHEK2* c.1100del PV, with a median age at diagnosis of 56 years. All tumors were of ductal histology, ER-positive, and poorly differentiated (grades 2 to 3). Another study also showed that *CHEK2* c.1100del male carriers had a lower mean age at diagnosis (53.8 years), which was statistically significant when compared to men without this PV [92]. 

The low-risk *CHEK2* c.470T>C (p.Ile157Thr) PV has also been described in association to MBC but with a low frequency and in a very limited number of studies [92]. 

### 5.4. ATM

The ataxia telangiectasia mutated (*ATM*) gene, located at 11q22.3, codifies a protein kinase with a key role in cell signaling in response to DNA double-stranded breaks and in recruiting proteins involved in repair. So far, *ATM* PVs have been associated with an autosomal dominant predisposition to breast, pancreatic, and prostate cancers [116]. In a large association study including only women, the presence of a protein-truncating variant in *ATM* was linked to an increased risk of BC (*p* < 0.0001), with an OR of 2.10 [117]. Data on *ATM* PVs and MBC is very scarce [118]. Recently, a study including patients referred for genetic testing due to personal and/or family histories of cancer found the risk of BC in men harboring an *ATM* PV to be 1.72 higher (95% CI, 1.08–2.75) [119].

### 5.5. Pathogenic Variants in Other Moderate to High-Risk Genes

Studies that applied MGPT in cohorts of MBC found PVs in new genes of lower frequency in addition to those previously described [25,92,97,120]. Most studies applied gene panels containing between a dozen to a hundred of cancer predisposing genes, including some with unknown association with MBC (Table 2). Nearly all of them identified *BRCA2* as the predominantly mutated gene, usually followed by *CHEK2*, *PALB2*, *BRCA1*, *ATM*, and *RAD51C*. Considering the two studies that were statistically powered in order to detect the magnitude of the associated risk, the following genes were related with increased risk of MBC: *BRCA2* (OR = 13.9), *CHEK2* (OR = 3.7), *PALB2* (OR = 6.6–17.3), and *RAD51D* (OR = 8.6–10.2) [92,120]. 

Further studies and case reports have already described PVs in other genes, such as *BRIP1* [121], *PTEN* [122], androgen receptor gene [123], *FANCM* [124], *NF1* [125], *MUTYH* [126], *MLH1* [127], and *PMS2* [95]. 

Overall, the detection rates of MGPT in MBC patients ranged from 9.0% to 31.8%, and the majority of cases are still named “sporadic”, since they do not present a clear familial clustering, probably resulting from the interaction between multiple and poorly penetrant alterations in other genes with environmental factors [128]. 

### 5.6. Low-Penetrance Variants and the Polygenic Risk

Genome-wide association studies (GWAS) indicate that low-penetrance variants also play a role in MBC risk, providing further information regarding the genetic basis of MBC. The largest GWAS study performed for MBC, which evaluated 1380 MBC cases and 3620 controls, identified three novel MBC predisposition loci (rs9371545 at 6q25.1 and rs78540526 and rs554219 at 11q13.3) and confirmed two previously associated regions (rs1022979 at 14q24.1 and rs35850695 at 16q12.1) [129,130]. Remarkably, these five loci are also associated with FBC, and there is evidence suggesting that up to 20% of confirmed FBC susceptibility single-nucleotide variants (SNVs) also influence the MBC risk [129]. In fact, a study from the Consortium of Investigators of Modifiers of *BRCA1/2* demonstrated that a polygenic risk score (PRS) based on the combined effects of 88 FBC susceptibility variants may provide an informative BC risk stratification in male carriers of *BRCA1/2* PVs [131]. In 2015, an Italian study including 386 MBC suggested that the 8q24.21 region is associated with MBC risk and showed that loci rs1562430/8q24.21 and rs1314913/14q24.1 significantly influence the BC risk in men [132]. Another Italian study suggested that *SULT1A1* p.Arg213His polymorphism (rs9282861) would act as a low-penetrance risk allele for MBC and would be associate with HER2 overexpression in tumors [133]. A polymorphism in the *CYP17* gene leading to increased serum levels of estrogen was initially suggested to be implicated in the development of MBC [134], but a larger recent study ruled out that possibility [135]. 

Recently, a large retrospective case-control study including over 150,000 women examined an 86-SNV PRS for *BRCA1*, *BRCA2*, *CHEK2*, *ATM*, and *PALB2* PV carriers [136]. The lifetime risk of FBC at 80 years ranged from the lowest PRS to the highest PRS, respectively, from 53.1% to 91.5% for *BRCA1* (gene-based risk of 73.5%), 50.8% to 94.2% for *BRCA2* (gene-based risk of 73.8%), 6.6% to 70.6% for *CHEK2* (gene-based risk of 22.1%), 12.9% to 58.3% for *ATM* (gene-based risk of 28.2%), and 26.2% to 79.2% for *PALB2* (gene-based risk of 50.1%). Even for non-carries, the lifetime risk of apparently sporadic FBC (gene-based risk of 12.7%) varied from a minimum of 2.5% for low-PRS to a maximum of 62.4%. As in FBC, the PRS potentially might modulate the risks of MBC, and a very high PRS could explain the heritability in apparently sporadic MBC testing negative for predisposition BC genes [129]. However, so far, we do not have enough data to calculate the male score with sufficient power to be useful in current clinical practice, but this is, undoubtedly, a field of intense research.

## 6. Multigene Panel Testing in MBC

Next-generation sequencing (NGS) has made possible the sequencing of multiple genes simultaneously through personalized multigene panels, which are now widely used in clinical practice following recommendations from cancer-specific guidelines [25,137]. In the FBC field, multigene panel testing has rapidly substituted *BRCA1/2*-only testing, since its use has shown to increase the detection rate of clinically relevant PVs in up to two-fold [138]. The wideness of the panel varies according to the country (up to an impressive amount of 100 cancer-associated genes) [139], and it seems that a specific small group of high-, moderate-, and low-penetrance genes can provide clinical benefits when included in a gene panel for BC predisposition in women [140]. 

The role of MGPT in genetic counseling for MBC patients is not established, and there is no formal recommendation regarding its use. At the present time, we do not have enough evidence to propose a specific gene panel for men with BC, because the associated risk of cancer for most candidate genes remains to be defined. A large Italian study applied a multigene custom panel of 50 cancer-associated genes in 503 *BRCA1/2* wild-type MBC patients, founding a germline PV in 5% of them, distributed in only 16 of the 50 genes evaluated [120]. Another study assessing the utility of MGPT in MBC found PVs in other genes than *BRCA1/2* that carry a higher risk for breast and ovarian cancers in women [92]. Although the correlation of most genes related to FBC is not clear when it comes to MBC risk, once MGPT in men with BC can also identify genes that have an impact on cancer prevention strategies for their relatives, especially women, it is reasonable to consider the application of small panels of selected genes in clinical practice. There is weak evidence linking the germline PVs in *TP53*, *CDH1*, and *PTEN* with a higher risk of BC in men, and the testing of such genes should be guided by the clinical characteristics of the associated syndromes (Li Fraumeni, Hereditary Diffuse Gastric Cancer, and Cowden, respectively) [92]. 

The clinical impact of multiple gene testing in MBC is still not clear, and probably, international cooperative efforts will be necessary to clarify this issue due to the rarity of the disease. Worries about MGPT use for men with BC are the same as those related to their application for women with BC, which can be added to the fact that knowledge of the germline PVs in MBC is scarcer and includes the absence of specific guidelines for the management of patients harboring PVs, the identification of multiple VUS with undefined clinical impacts, and patient harm due to anxiety by the findings, in addition to the financial impact on health systems [141].

**Table 2:** Studies performing multigene panel testing in MBC patients.

Author, Year	Cohort	Panel Size	Detection Rate	Gene with PV (%)	Observations
Tedaldi et al. 2020 [97]	70 patients selected from genetic counselling	94 genes	21.4%	*BRCA2* (8.6), *BRCA1* (4.3), *PALB2* (1.4), *CHEK2* (1.4), *ATM* (1.4), *RAD51C* (1.4), *BAP1* (1.4), *EGFR* (1.4)	Two patients (2.9%) had a second contralateral MBC, and 16 (22.9%) had a second non-BC malignancy. Twenty-four patients (34.3%) had first- and/or second-degree relatives with BC/OC, and 17 patients (24.3%) had first- and/or second-degree relatives with other cancers. Three patients (4.3%) had a FH of MBC among first-degree relatives.
Gaddam et al. 2020 [96]	414 men who underwent MGPT for a variety of clinical indications. Eighteen patients had PH of BC.	Several commercial panels	27.8%	*BRCA2* (16.7), *NBN* (5.6), *BARD1* (5.6) ^a^	
Scarpitta et al. 2019 [95]	81 patients selected from genetic counselling	24 genes involved in breast and ovarian cancer predisposition, maintenance of genome stability and DNA repair	18.5%	*BRCA2* (13.6), *BRIP1* (2.5), *MUTYH* (1.2), *PMS2* (1.2)	Twelve patients developed BC before 50, 10 had a diagnosis of another primitive cancer, and 1 had a bilateral BC. The most common additional cancer was prostate cancer, with a 40% (4/10) frequency rate. In this cohort, 37% (30/81) reported to have BC/OC history among first-degree relatives.
Rizzolo et al. 2019 [120]	523 patients, unselected for age at diagnosis and FH of cancer from 13 Italian Investigator Centers	50 genes	9.0%	*BRCA2* (2.9), *PALB2* (1.1), *BRCA1* (1.0); *ATM* (0.6); *APC* (0.4), *BARD1* (0.4), *BLM* (0.4), *CHEK2* (0.4), *FANCM* (0.2), *RAD51D* (0.4), *CASP8* (0.2), *EPCAM* (0.2), *MUTYH* (0.2), *NF1* (0.2), *RAD50* (0.2), *RAD51C* (0.2)	This study included 80 cases with no prior *BRCA1/2* testing and 443 cases negative for *BRCA1/2*. Eighty-seven (16.7%) had first-degree FH of BC/OC and 230 (44.1%) of any cancer. PH of other cancers, mostly prostate, colorectal, and bladder cancer, was observed in 99 cases (18.9%).
Fostira et al. 2018 [94]	102 patients unselected for FH and age at diagnosis	94 genes	12.7%	*BRCA2* (6.9), *ATM* (2.0), *BRCA1* (1.0), *CHEK2* (1.0), *PMS2* (1.0), *FANCL* (1.0)	Fifteen percent (15/102) of the patients were diagnosed with a second primary cancer, of which colorectal and duodenal cancer represented one-third of them. Other cancer types involved are prostate, thyroid, pancreatic, bladder, laryngeal, and non-Hodgkin’s lymphoma. Two of these patients were diagnosed with a metachronous BC.
Vogel Postula et al. 2018 [93]	381 patients	8 to 32 genes	12.1%	*BRCA2* (5.5); *CHEK2* (4.5); *PALB2* (1.0); *BRCA1* (1.0); *ATM* (0.5) ^b^	Three hundred and fifteen patients with no prior *BRCA1/2* testing and 66 negatives for *BRCA1/2.*
Pritzlaff et al. 2017 [92]	708 patients (538 Caucasian or Ashkenazi Jewish)	16 genes	18.1%	*BRCA2* (8.1), *CHEK2* (3.8), *ATM* (1.0), *BRCA1* (0.9), *PALB2* (0.8), *NF1* (0.6), *BARD1* (0.4), *BRIP1* (0.2), *MRE11A* (0.2), *NBN* (0.2), *RAD51D* (0.2)	The study included 551 cases with no prior *BRCA1/2* testing and 197 cases negative for *BRCA1/2.* Four percent of MBC patients had a second primary BC, and additional non-breast primary cancers were reported for 23.4%. The most common additional cancer was prostate cancer (9.5%). A FH of MBC was reported for 6.4% of patients.
Susswein et al. 2016 [91]	10,030 individuals (men and women) referred for evaluation by an NGS hereditary cancer panel. Included 51 men with history of BC.	Several commercial panels (up to 29 genes)	11.8%	*CHEK2* (7.8), *BRCA2* (2.0), *PALB2* (2.0), *BRCA1* (2.0)	
Tung et al. 2015 [13]	2158 individuals (men and women). Included 22 men with a PH of BC.	25 genes	31.8%	*BRCA2* (13.6); *CHEK2* (4.5); *PALB2* (4.5); *BRCA1* (4.5); *ATM* (4.5) ^c^	The study included 1781 individuals who were referred for commercial *BRCA1/2* testing and 377 individuals who previously tested negative for *BRCA1/2* mutations through an academic high-risk program

FH: family history; =, BC: breast cancer, OC: ovarian cancer, PH: personal history, and NGS: next-generation sequencing. ^a^—Data refers only to the 18 patients with BC history, ^b^—other genes not reported, and ^c^—data refers only to the 22 men with BC history.

## 7. Effect of Family and Personal History on MBC Risk Estimates

As stated before, a family history of BC in a first-degree relative constitutes an important risk factor for MBC [142,143]. The first report suggesting an association between family history of BC and increased risk of MBC was probably made by Williams in 1889 [144]. In a retrospective cohort with 100 cases of MBC, three out of 29 had a first- or second-degree relative with BC [144]. During the following decades, other studies also demonstrated this association [145,146,147]. 

Currently, it is known that having a first-degree relative with BC confers a two- to three-fold increase in the risk of having MBC [20]. Population-based studies have shown that about 20% of all MBC patients have a history of BC in a first-degree female relative, which is higher than that observed in women (~7%) [148]. Interestingly, having a brother with BC increases the risk of a woman having the disease by 2.48-fold, compared to a 1.87-fold increase with a sister diagnosed with BC [149]. 

A family history of other malignancies is also linked to increased risk of MBC. Approximately 15–20% of men with BC reported a family history of breast or ovarian cancer [12]. A study by Brinton et al. [150] prospectively assessed 324,920 men, among whom 121 developed BC. They showed a 10-fold increase in the risk of BC in men with a sister and a mother with BC (OR 9.73; 95% CI 3.96–23.96) but, also, an increased risk if a family history of other cancers was reported (OR 1.26; 95% CI 0.86–1.83), especially for prostate cancer (OR 1.51; 95% CI 0.88–2.61). 

It is important to note that most of the studies analyzing the association between family history of BC and MBC risk did not determine the germline variant status of the patients. Distinctly, Calip et al. [151] recently published a case-control study aiming to characterize the association of family history of BC with a diagnosis of MBC in 3647 men who were *BRCA*-negative in a commercial testing for germline PVs that included 25 cancer susceptibility genes. They showed that men with a first- or second-degree relative with BC had more than four-fold increase in the risk of a BC diagnosis (OR 4.7; 95% CI 4.1–5.3) compared to those who did not have a BC family history. There was also an increased risk of MBC among men with a first- or second-degree male relative diagnosed with BC (OR 17.9; 95% CI 7.6–42.1), as well as among those with both male and female relatives affected (OR 15.7; 95% CI 4.4–55.3). 

It is expected that men with a previous diagnosis of BC have an elevated risk for recurrence or a second BC, independently of whether a known genetic or environmental risk factor is identified. In a cohort of 1788 men with a first primary BC registered in the SEER program, who were unselected for other risk factors and not subjected to genetic testing, investigators found that the risk of developing a contralateral second BC was increased by nearly 30-fold compared to average men, with the highest risk seen in men diagnosed before the age of 50 years (increased by 110-fold) [49]. In a Swedish nation-wide study, the risk of a BC was found to be 93-fold higher in men with a personal history of a first BC, compared to women (3.2-fold) [152]. For men diagnosed with BC, the risk of recurrence continues during the following 15 years and beyond [4,152].

## 8. MBC and Other Cancer Risks

Evidence suggests that MBC is associated with an elevated risk of second malignancies other than BC. A multicenter study with unselected men with BC showed a 34% overall excess risk of second primary malignant tumor following the diagnosis of MBC [153]. The most frequent diagnosed cancers were small intestine (standardized incidence ratio (SIR) = 4.95, 95% CI, 1.35–12.7), pancreatic (SIR 1.93, 1.14–3.05), rectum (SIR 1.78, 1.20–2.54), nonmelanoma skin tumors (SIR 1.65, 1.16–2.29), prostate (SIR 1.61, 1.34–1.93), and lymphohematopoietic system (SIR 1.63, 1.12–2.29). No significant increase of prostate cancer (SIR 1.09, 95% CI 0.85–1.37) in patients with a primary MBC was seen in the report by Auvinen et al. [49], but they found an increased risk of melanoma (SIR 2.41, 95% CI 1.15–4.43). The fact that the occurrence of other tumors than BC is increased in families with MBC patients suggests that MBC also shares susceptibility factors with other cancers [154].

In male *BRCA1/2* PVs carriers, prostate cancer is the most commonly diagnosed cancer [155]. They have an increased risk of prostate cancer before the age of 65 years and a worse prognosis. Consequently, it is reasonable for men with germline *BRCA1/2* PVs to consider beginning shared decision-making PSA screening at 40 years of age at annual intervals rather than every other year. MRI has generally been shown to have superior sensitivity for clinically significant sporadic prostate cancer when compared to TRUS biopsies, but no data is available for carriers of *BRCA1/2* [156].

In the near future, male carriers of *BRCA1/2* PVs should benefit from PRS cancer risk stratification that might enable these men and their physicians to make informed decisions on the type and timing of breast and prostate cancer risk management. For example, the prostate cancer risk by age 80 years at the 5th and 95th percentiles of the PRS varies from 7% to 26% for carriers of *BRCA1* PVs and from 19% to 61% for carriers of *BRCA2* PVs, respectively [131]. 

Emerging data have suggested a potential benefit of pancreatic cancer screening in selected individuals at increased risk, but a lot of controversy remains on this topic. Pancreatic cancer screening recommendations are discussed if a PV is identified in a pancreatic cancer gene (*ATM, BRCA1, BRCA2, CDKN2A, MLH1, MSH2, MSH6, EPCAM, PALB2, STK11*, and *TP53*) in a patient with a family member (first or second degree) with a history of pancreatic cancer [157]. Due to the lack of definitive data on the long-term screening results, this decision requires a discussion with the patient of the risks and benefits. 

The risk of other malignancies is also increased in male *BRCA1/2* carriers, such as bladder, colorectal, head and neck, lung, nonmelanoma skin, and leukemia, among others. The absence of reliable risk estimates in *BRCA1/2* PV carriers for these cancers, especially for colorectal cancer, leads to uncertainty about the appropriate screening protocols [131]. 

## 9. Screening and Surveillance of High-Risk Men

In contrast to women, for whom screening mammography has proved to play a role in reducing BC mortality [158,159], there is no recommendation for general screening in MBC detection due to the overall low prevalence of the disease [160]. Even in men with known risk factors, the value of such strategy has not been established [161]. 

Previous studies in the diagnostic setting of men with suspected breast lesions show the sensitivity, specificity, and negative predictive values approaching 100% for mammography and ultrasonography [162,163,164]. A large retrospective study by Gao et al. [165] reviewed 2052 breast imaging examinations of 1869 men, of whom 271 (13.2%) underwent screening mammography due to personal or family history of BC and/or genetic mutations. They showed mammographic screening sensitivity, specificity, and positive predictive value of the biopsy of 100%, 95%, and 50%, respectively. In another study, Marino et al. [166] aimed to investigate the utility of a mammography for BC screening in a cohort of men at increased risk for BC, defined as having familial and/or personal history of BC and/or a known germline PV in *BRCA*. They showed a cancer detection rate of 4.9/1000 in that population, which was quite similar to that of the screening mammography in women (5.4/1000), concluding that a screening mammography is useful and should be performed in men at an increased risk for BC. 

Currently, there are no published prospective large cohorts of high-risk men validating such results. As a consequence, for men harboring *BRCA1/2* PVs, despite the increasing knowledge on gene-specific cancer phenotype differences to guide surveillance programs [155], there is no consensus on the best follow-up strategy for these individuals, and guidelines diverge based on recommendations (Table 3). 

For patients with Klinefelter syndrome, routine screening mammography is not an endorsed recommendation [167]. The European Academy of Andrology and the European Society of Endocrinology suggest clinical breast and axilla examinations every two years in adult patients with KS and an eventual mammography and/or breast ultrasonography, especially in those patients with a family history of BC or a suspicious lesion in the breast [168].

**Table 3:** Main recommendations of the selected guidelines on the surveillance of male carrier of *BRCA1* and *BRCA2* PVs.

Guideline (Year)	Recommendations
NCCN (2021) [157]	Clinical breast exam, every 12 months, starting at age 35 years Consider annual mammogram screening in men with gynecomastia starting at age 50 or 10 years before the earliest known MBC in the family (whichever comes first) Starting at age 40 years: recommend prostate cancer screening for *BRCA2* carriers, and consider prostate cancer screening for *BRCA1* carriers Melanoma risk management is appropriate, such as annual full body (examination and minimizing UV exposure) Consider pancreatic cancer screening beginning at age 50 years (or 10 years younger than the earliest exocrine pancreatic cancer diagnosis in the family, whichever is earlier) for individuals with exocrine pancreatic cancer in ≥1 first- or second-degree relatives from the same side of the family as the identified germline PV
ASCO (2019) [4]	Annual mammogram may be offered to men with a history of breast cancer and a genetic predisposing mutation
SEOM (2019) [169]	No evidence of clinical benefit of breast screening. Consider mammography in the case of gynecomastia Annual screening with PSA for prostate cancer from the age of 40 years (recommended in *BRCA2*, and offer in *BRCA1*) Consider pancreatic cancer surveillance with EUS and MRI in carriers with a first-degree relative with pancreatic cancer from the age of 50 or 10 years before the youngest diagnosis in the family Consider skin and eye examination for melanoma screening according personal/familiar risk factors
ESMO (2016) [170]	Annual clinical breast examination by a physician, starting from the age of 30. No evidence exists to justify or support routine annual breast imaging among male carriers Annual screening for prostate cancer may be considered from the age of 40, particularly for BRCA2 carriers BRCA2 carriers may consider annual skin and eye examination as screening for melanoma BRCA2 carriers may consider annual screening for pancreatic cancer with EUS or MRI/MRCP while being informed that data supporting this approach is very limited. There is no consensus when screening should commence; however, age 50 or 10 years before the earliest diagnosed case in the family would be reasonable
ICR (2015) [171]	No breast surveillance for male carriers No surveillance is recommended for other cancers

NCCN: National Comprehensive Cancer Network, ASCO: American Society of Clinical Oncology, SEOM: Spanish Society of Medical Oncology, ESMO: European Society for Medical Oncology, and ICR: Institute of Cancer Research.

## 10. Implications for Systemic Treatment

Knowledge of the germline mutational status in MBC might have important therapeutic implications, with an impact on survival. Due to the mechanism of action of platinum compounds, which create DNA crosslinks and consequent double-stranded breaks [172], *BRCA1/2*-related BC are known to present a significant response to platinum-based chemotherapy [173]. HRR is the major cell surveillance mechanism of DNA double-stranded break repair necessary for genome integrity after platinum damaging, mainly triggered by functional HRR genes such as *BRCA1/2*, among others. The phenotype of deficiency of homologous recombination (HRD) is of clinical relevance, as it is indicative of a sensitivity to targeted therapy with poly adenosine diphosphate-ribose polymerase inhibitors (PARPi), as well as to DNA damaging reagents such as platinum compounds [174].

Although no study has assessed the HRD phenotype specifically in MBC, for patients with a germline *BRCA1/2* PV with metastatic BC, platinum-based chemotherapy should be prioritized [175]. Furthermore, PARPi are currently a therapeutic option for BC patients harboring germline *BRCA1/2* PVs in the metastatic setting [176,177]. PARPi disrupt the DNA single-stranded break repair pathway, making the cell dependent on the homologous recombination mechanisms to be repaired. In tumors with impaired homologous recombination, such as those associated with *BRCA1/2* PVs, the absence of both mechanisms leads to cell death (synthetic lethality) [178]. In men with prostate cancer who harbor germinal PV in *BRCA1/2* and in other DNA damage response-altered genes, PARPi have also shown antitumoral activity, with a safe toxicity profile [179,180,181]. 

## 11. Conclusions

Although the main predisposing genes to FBC are also those more frequently found in MBC, their prevalence and phenotypic characteristics differ in these two populations. It is possible that other gender factors play a role in BC carcinogenesis.

The contribution of germline alterations to BC risk in men is significant, and physicians need to be aware and refer these patients for genetic counseling. The role of broad genetic panels in this scenario is still unclear, and it is likely that smaller panels with selected genes can warrant comprehensive coverage in the germinal evaluation of MBC patients. 

Population studies to estimate the lifetime risk of BC associated with the presence of different mutations and to establish the best strategy of screening for men harboring those variants are necessary. 

## Data Availability

Not applicable.
